# Supplementary material for: Transcriptome Comparison between Two Strains of Ustilago esculenta during the Mating
Source: J Fungi (Basel). 2022 Dec 23;9(1):32. doi: 10.3390/jof9010032 (PMC9862937; doi:10.3390/jof9010032)
Supplement: Supplementary file 1 [file jof-09-00032-s001.zip › Supplement data.pdf]

Table S1. Strains used in this study.

Strain name	Brief description	Resistence	Reference
Ue_T14	Wild type strain of UeT14	/	Zhang,Yafen[24]
Ue_T55	Wild type strain of UeT55	/	Zhang,Yafen
Ue_MT46	Wild type strain of UeMT46	/	Zhang,Yafen
Ue_MT10	Wild type strain of UeMT10	/	Zhang,Yafen
Ue_T14△*Ueg943*	*g943* deletion in UeT14	Hygromycin B	This study
Ue_T55△*Ueg943*	*g943* deletion in UeT55	Hygromycin B	This study
Ue_T14△*Ueg4344*	*g4344* deletion in UeT14	Hygromycin B	This study
Ue_T55△*Ueg4344*	*g4344* deletion in UeT55	Hygromycin B	This study
Ue_T14△*Ueg1072*	*g1072* deletion in UeT14	Hygromycin B	This study
Ue_T55△*Ueg1072*	*g1072* deletion in UeT55	Hygromycin B	This study
Ue_T14△*Ueg4195*	*g4195* deletion in UeT14	Hygromycin B	This study
Ue_T55△*Ueg4195*	*g4195* deletion in UeT55	Hygromycin B	This study
Ue_T14△*Ueg4740*	*g4740* deletion in UeT14	Hygromycin B	This study
Ue_T55△*Ueg4740*	*g4740* deletion in UeT55	Hygromycin B	This study
Ue_T14△*Ueg5676*	*g5676* deletion in UeT14	Hygromycin B	This study
Ue_T55△*Ueg5676*	*g5676* deletion in UeT55	Hygromycin B	This study
Ue_T14△*Ueg6161*	*g6161* deletion in UeT14	Hygromycin B	This study
Ue_T55△*Ueg6161*	*g6161* deletion in UeT55	Hygromycin B	This study
WT	UeT14 and UeT55 cross	/	Zhang,Yafen
Δg943	UeT14*Δg943* and UeT55*Δg943* cross	Hygromycin B	This study
Δg4344	UeT14*Δg4344* and UeT55*Δg4344* cross	Hygromycin B	This study
Δg1072	UeT14*Δg1072* and UeT55*Δg1072* cross	Hygromycin B	This study
Δg4195	UeT14*Δg4195* and UeT55*Δg4195* cross	Hygromycin B	This study
Δg4740	UeT14*Δg4740* and UeT55*Δg4740* cross	Hygromycin B	This study
Δg5676	UeT14*Δg5676* and UeT55*Δg5676* cross	Hygromycin B	This study
Δg6161	UeT14*Δg6161* and UeT55*Δg6161* cross	Hygromycin B	This study

Table S2. The quality evaluation for transcriptome sequencing data.

Sample name	Raw reads	Clean reads	Clean bases	Error rate(%)	Q20(%)	Q30(%)	GC content(%)
T_0h_1	63028620	61828626	9.27G	0.03	97.41	93.29	57.73
T_0h_2	58465256	56911444	8.54G	0.02	98.02	94.83	56.9
T_0h_3	61717374	60387654	9.06G	0.02	98.11	94.98	57.71
T_24h_1	51982916	50884824	7.63G	0.02	98.06	94.79	57.47
T_24h_2	63126784	62101672	9.32G	0.02	97.97	94.61	57.45
T_24h_3	58069690	56615754	8.49G	0.02	98.02	94.79	57.59
T_36h_1	65575728	64566864	9.69G	0.03	97.88	94.39	57.38
T_36h_2	58135734	57011646	8.55G	0.03	97.78	94.24	57.48
T_36h_3	57233694	56133656	8.42G	0.02	98.03	94.79	57.45
T_48h_1	57646070	56654842	8.5G	0.02	98.04	94.79	57.51
T_48h_2	63453004	62290970	9.34G	0.02	98.05	94.81	57.41
T_48h_3	59929234	58029586	8.7G	0.02	98.11	95	57.5
T_60h_1	57738162	56761712	8.51G	0.02	98.11	94.96	57.43
T_60h_2	58035490	56884260	8.53G	0.03	97.32	92.92	57.31
T_60h_3	49590524	48484326	7.27G	0.02	97.96	94.67	57.37
MT_0h_1	72564574	70807538	10.62G	0.02	97.98	94.67	57.63
MT_0h_2	60399364	59153946	8.87G	0.02	98.04	94.79	57.62
MT_0h_3	66734618	65241168	9.79G	0.02	98.19	95.13	57.71
MT_24h_1	59926520	58762080	8.81G	0.02	98.03	94.78	57.55
MT_24h_2	67568086	66342040	9.95G	0.02	97.96	94.61	57.51
MT_24h_3	66903844	65777874	9.87G	0.02	97.94	94.54	57.43
MT_36h_1	52764300	51781094	7.77G	0.02	98.27	95.36	57.45
MT_36h_2	59412234	58027264	8.7G	0.02	98.03	94.79	57.48
MT_36h_3	47897528	46808134	7.02G	0.02	98.15	95.08	57.45
MT_48h_1	67878802	66064674	9.91G	0.02	98.05	94.87	57.31
MT_48h_2	70880600	69455894	10.42G	0.03	97.78	94.18	57.33
MT_48h_3	55946556	54882000	8.23G	0.03	97.83	94.33	57.55
MT_60h_1	52010644	50872250	7.63G	0.02	98.12	95.01	57.36
MT_60h_2	62363934	60532096	9.08G	0.02	98.08	94.94	57.18
MT_60h_3	66081768	64332542	9.65G	0.02	97.93	94.53	57.51

Table S3. Primers used in this study.

Primer	Sequence (5′→ 3')	Purpose
g943-cF	GGCATCCTCCTTGGGGCAATGG	
g943-cR	ATGAAGTTCGCCTTCGGTACCCTTGTCTG	Clone
g4344-cF	ATGAAGATCCAGGCCTCTTTCGTGAC	
g4344-cR	GTAGGACACGCCCCCGCCGGAAGTCTC	
g1072-cF	ATGCAGAATTTCCTCGTTCTCCTCCC	
g1072-cR	GGAGGTCAAAGGCTGG	
g4195-cF	ATGGTCAAGCTTCAGCAGTG	
g4195-cR	GTTCTGGACGGCGTCTTTCTTGTCG	
g4740-cF	ATGGCTTCGAAATCAGACCGAAGAGG	
g4740-cR	GAACTGGGCCGAGAGGAAAATTGG	
g5676-cF	ATGACCATCAAAGTCAGCCCTATTGCC	
g5676-cR	ATCGTGGTAGTGCTTCTCAGGGTCCTTGG	
g6161-cF	ATGGTCGGCGCTAAGCTGG	
g6161-cR	TTAGCGACGACGGCGAGAG	
g943-UF1	ACTTTACGGGAGCGGGGTACCCACTACTACGATCATTGCTTCAAGC	
g943-UR1	TCTAGAGGATCCCCGGGTACCCCGCTCCCGTAAAGTCACAA	
g943-DF1	ACCTGCAGGCATGCAAGCTTCTTTCGGCGTTTTGGGACA	
g943-DR1	AGGGTGGAAGGGATTGAAGCTTGGCGTAATCATGGTCATA	
g4344-UF1	GAGAGCACACGCATCGGTACCTAGCTTCAGCGTTGAGCTCGA	Delection mutant
g4344-UR1	TCTAGAGGATCCCCGGGTACCGATGCGTGTGCTCTCTTTTCCG	construction and
g4344-DF1	GACCTGCAGGCATGCAAGCTTGCAAGTCAAAGGTCACGTATTCTACA	Verifycation
g4344-DR1	TGACCTTTGACTTGCAAGCTTTTACGGTTTCTCACAGCATCAAGT	
g1072-UF1	AGTGAATTCGAGCTCGGTACCGGGTGATGTTGGTATCTTGGAATT	
g1072-UR1	TCTAGAGGATCCCCGGGTACCCTGGACGGTGTGCTGGTTACG	
g1072-DF1	GACCTGCAGGCATGCAAGCTTCCTGTCCATCTTCCGCCC	

g1072-DR1	GACCATGATTACGCCAAGCTTCCGCAGGCTTTCGCCCAG	
g4195-UF1	AGTGAATTCGAGCTCGGTACCGCACGTCCCCAACCACACA	
g4195-UR1	TCTAGAGGATCCCCGGGTACCTGAAAGCCCTACCTTATCCACC	
g4195-DF1	GACCTGCAGGCATGCAAGCTTCGAAGCGAGGTGAGGAGGG	
g4195-DR1	GACCATGATTACGCCAAGCTTTCCACCAAGACATGTGCTTGC	
g4740-UF1	AGTGAATTCGAGCTCGGTACCGCACGTCCCCAACCACACA	
g4740-UR1	TCTAGAGGATCCCCGGGTACCTGAAAGCCCTACCTTATCCACC	
g4740-DF1	GACCTGCAGGCATGCAAGCTTCGAAGCGAGGTGAGGAGGG	
g4740-DR1	GACCATGATTACGCCAAGCTTTCCACCAAGACATGTGCTTGC	
g5676-UF1	AGTGAATTCGAGCTCGGTACCCCTCGCCCCCTCGCTCCA	
g5676-UR1	TCTAGAGGATCCCCGGGTACCGCTGAAGTCCGGGTGGTCA	
g5676-DF1	GACCTGCAGGCATGCAAGCTTTATAATTAATTTCTATTAATTTTCAAGTCTTCTG	
g5676-DR1	GACCATGATTACGCCAAGCTTTGTGATGAAGTAATTTGCATAGGCA	
g6161-UF1	TTCGAGCTCGGTACCTTCAGAATCGGCTCTGGCTC	
g6161-UR1	GAGGATCCCCGGGTACCGTACTCCCTCCCTAAACCAT	
g6161-DF1	TCGACCTGCAGGCATGCAAGCTTTGGGCAGAGAGAAAAGGGGA	
g6161-DR1	ATGACCATGATTACGCCAAGCTTACATATCAGCTCCCGCTCGA	
g943-QF	TGCAAGTGGAGGAGCTACAAC	
g943-QR	GCTTGGACCCAGTAGCTGTCGTGCTCATC	
g4344-QF	CGCTCAAACCAACTCCGATG	
g4344-QR	AGCCGTCGTCACTGTTCTGC	
g1072-QF	CAGAATTTCCTCGTTCTCCTCCCTCTTG	
g1072-QR	TGAACCTCGCCGTTCTTCCAGTCG	
g4195-QF	ATGATGGTACTGACTCCGGTCGTGGCGACTCC	q-PCR
g4195-QR	AGTCTTCAGAGCAGAAGCCGGTGGTTCG	
g4740-QF	ACGCCATGGTCATCATGGTCTCG	
g4740-QR	GGTCAGGCAGTTGAGGAGCATCAGCATCATC	
g5676-QF	CACGCACTTCGGATTTGAGTGTAGCATGG	
g5676-QR	CCGCCTTCCCTGAACGAGAACTTCTTCC	

g6161-QF	CAATGGTTCGGGAATGTGC
g6161-QR	GGGATACTTGAGCGTGAGGA

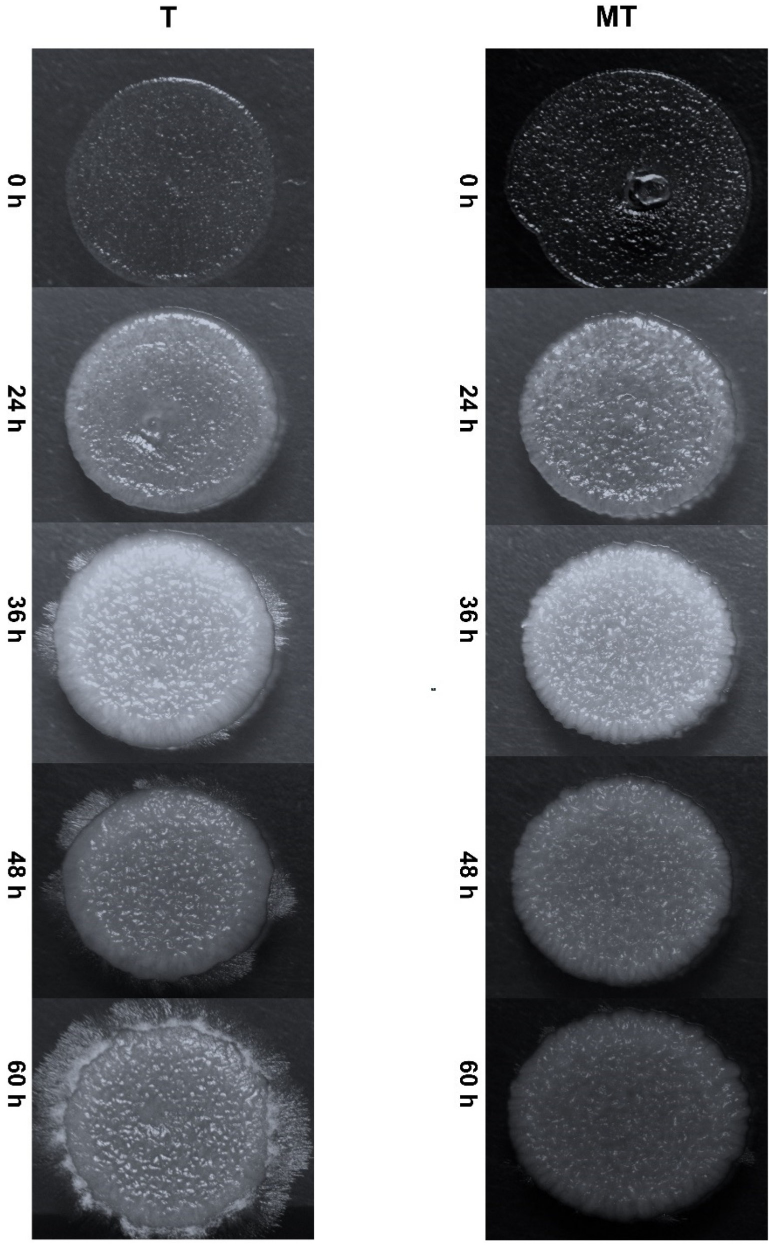

Figure S1. Mating and filamentous growth of the T- and MT-type *U. esculenta*. T-type mating strain was hybridized by the compatible T14 strain and T55 strain, and MT type mating strain was hybridized by the compatible MT10 strain and MT46 strain. They were grown on YEPS medium. Take photos at 0 h, 24 h, 36 h, 48 h and 60 h under stereo microscope.

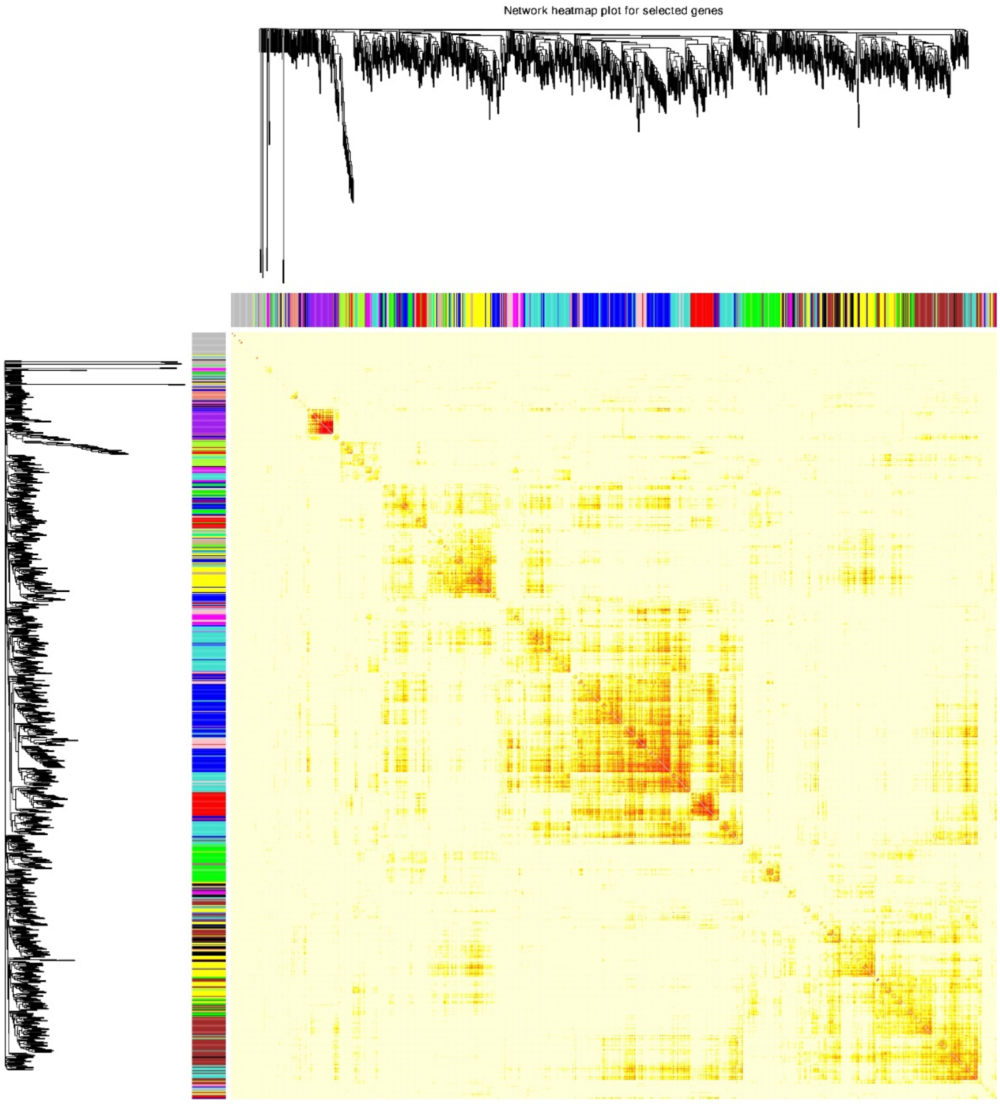

Figure S2: The overall relevance of different modules.

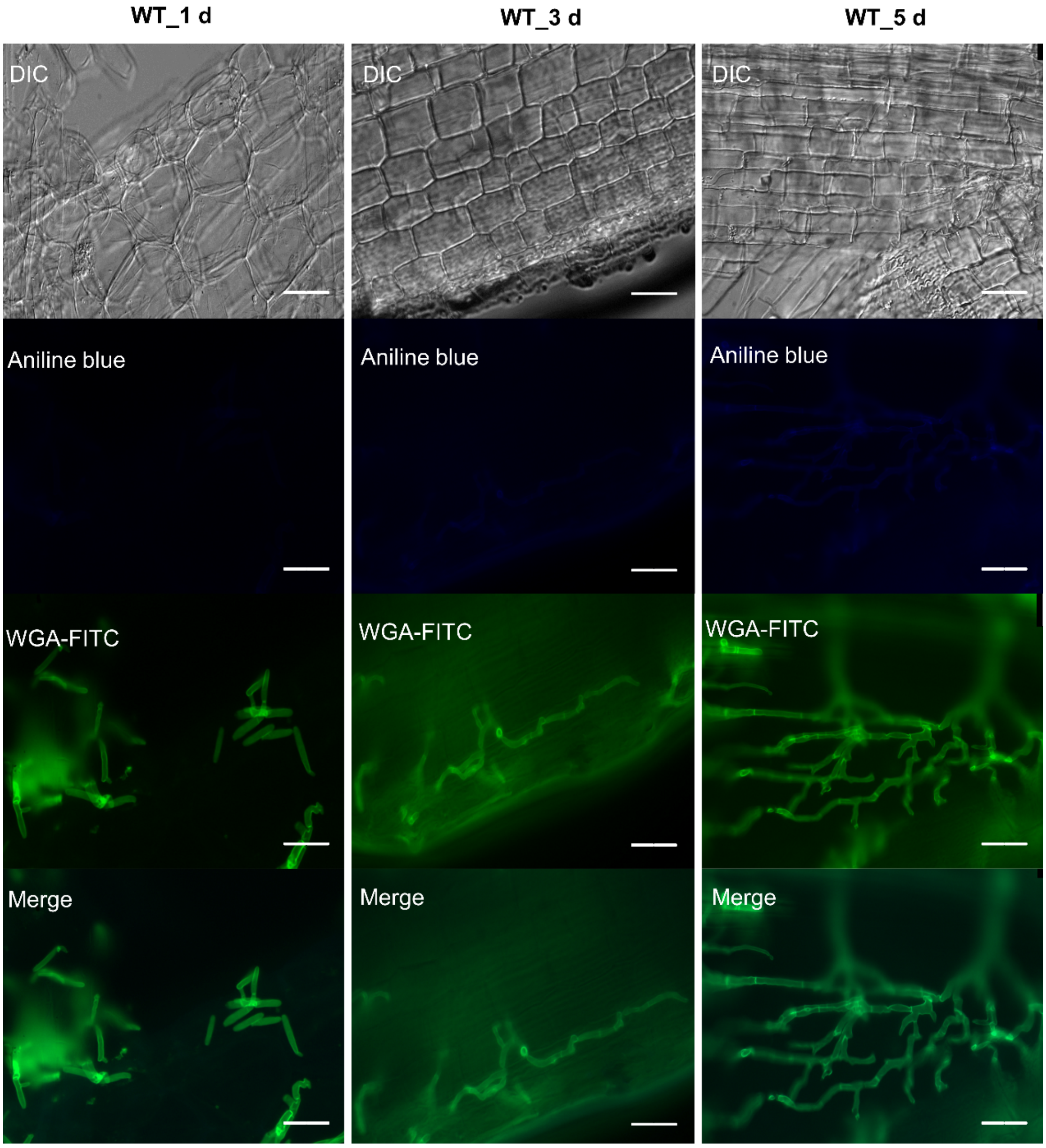

Figure S3. The infection of T-type *U. esculenta* did not induce callose deposition. Photomicrographs were taken 1, 3, and 5 dpi post infection (dpi). The WT strain does not induce callose deposition. The callose was stained by Aniline blue. It is excited at 405 nm, and the fluorescence was detected at 440 to 460 nm. The mycelium of U. esculenta is stained by WGA-FITC. It is excited at 488 nm, and the fluorescence was detected at 495 to 530 nm. Plant cell structure is detected by DIC. Merge image shows the superimposition of mycelium display image and aniline blue deposition image. The scale bar represents 62.5 μm.

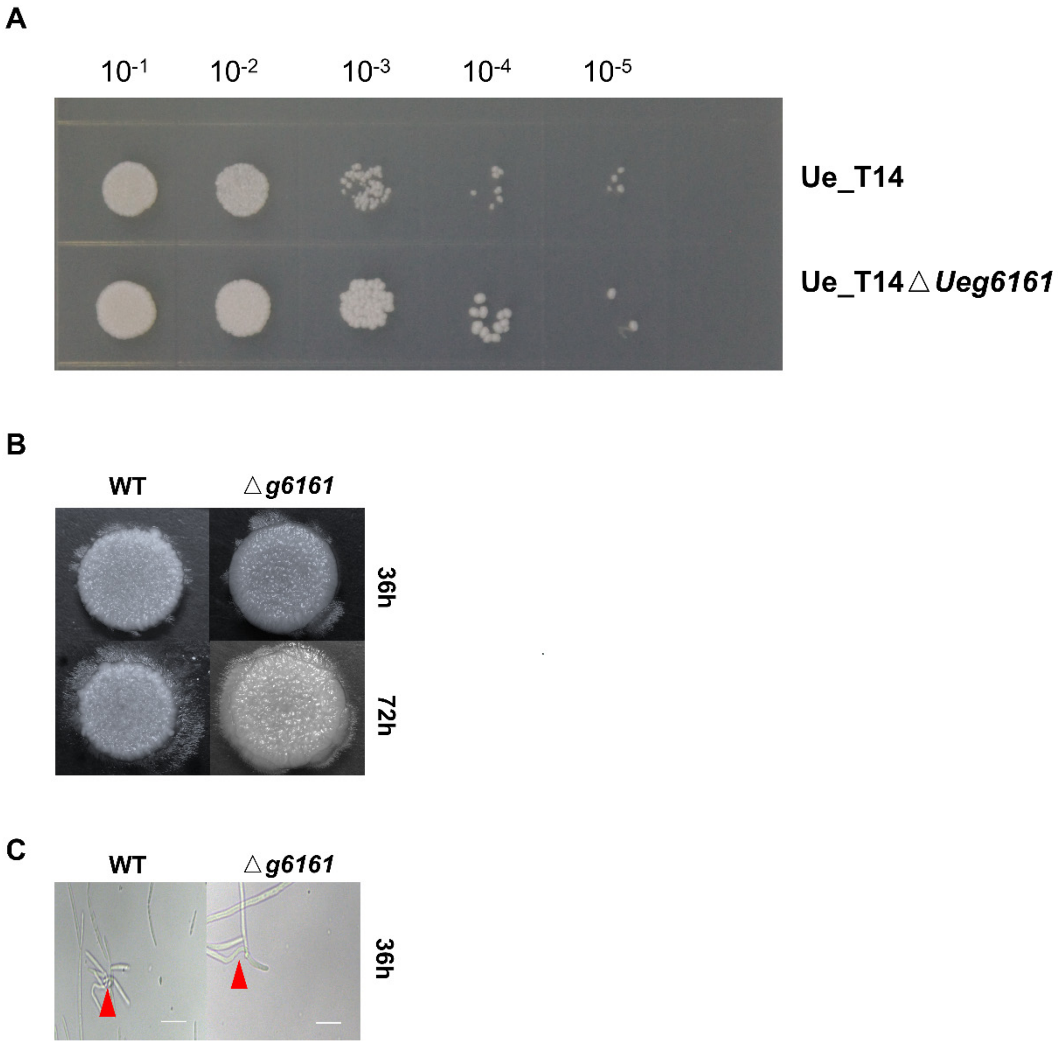

Figure S4. Effects of *g6161* deletion on haploid growth and mating. They were grown on YEPS medium. Photographs were taken at 72 h under at 36 h under a stereo microscope. UeT14 and UeT55 cross. UeT14Δ*g6161* and UeT55Δ*g6161*cross. (A) Deletion of *g6161* didn't impair haploid growth. Photograph the 72 h haploid growth state with camera. (B) Compared with WT, mating and filamentous growth of the gene deletion of *g6161*. Deletion of *g6161* didn't impair hypha mating.（C）Compared with the WT type, Forming state of the conjugation tube of the gene deletions of *g6161* at 36 h. Deletion of *g6161* didn't impair forming of the conjugation tube. (forming of conjugation tube pointed by red arrow). The scale bar represents 62.5 μm in 36 h.

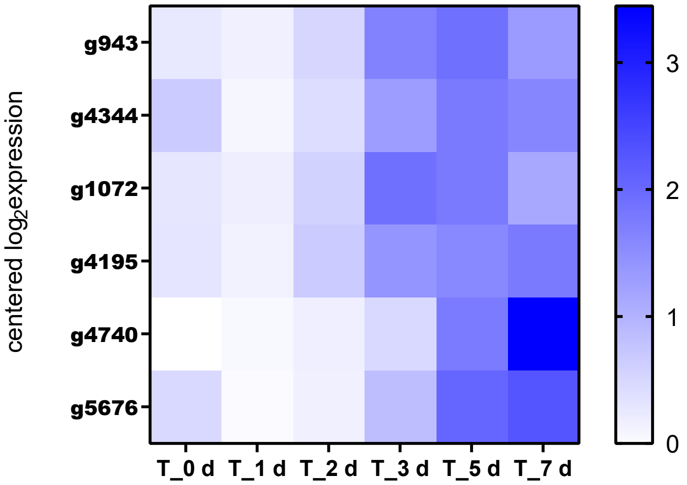

Figure S5. Gene expression pattern during the infection. The heat map shows the expression profi les of the expression of genes. Log2 expression levels are visualized relative to the mean expressio n across 0 d,1 d,2 d,3 d,5 d,7 d stages of T-type *U. esculenta*.

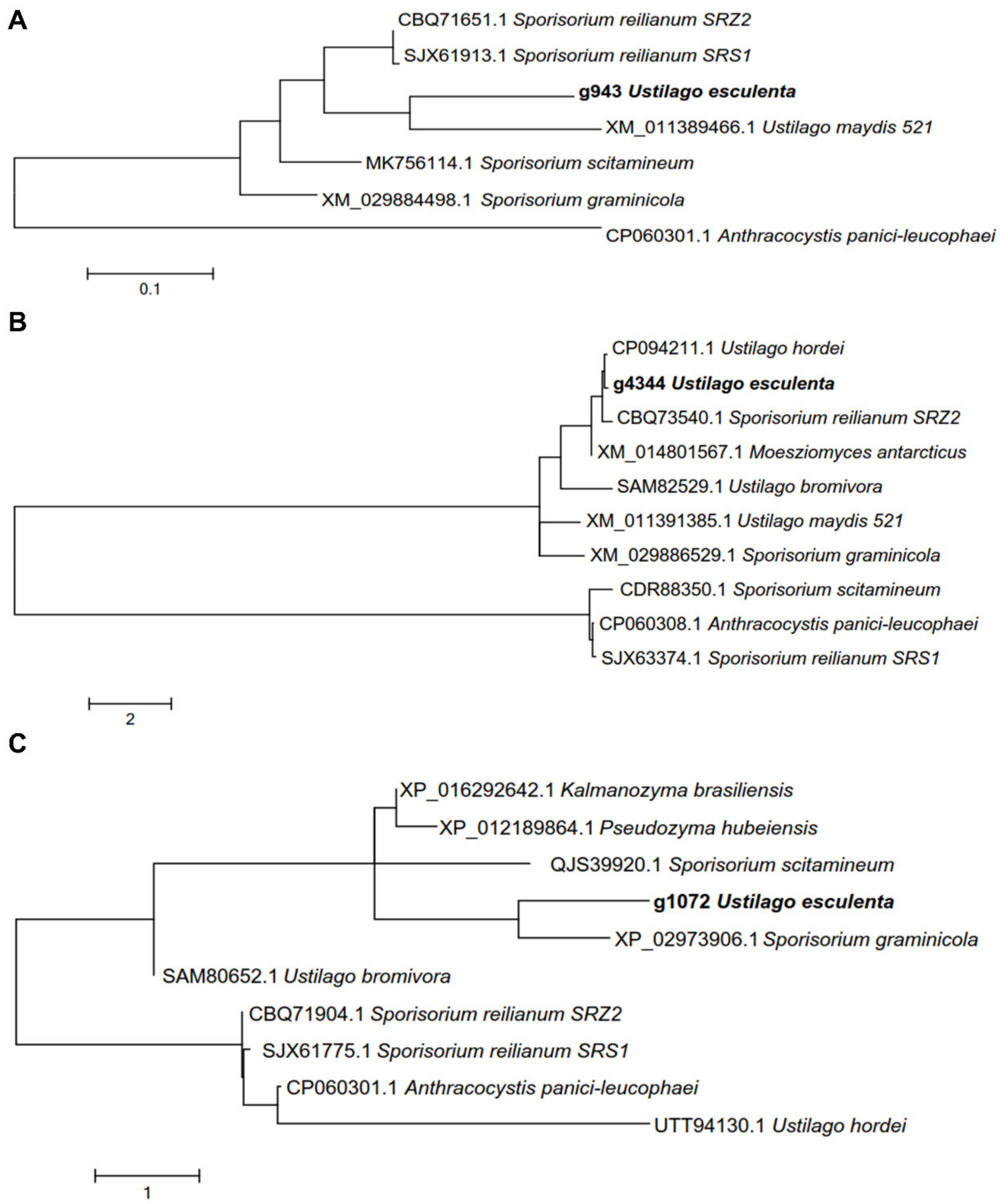
A
CBQ71651.1 Sporisorium reilianum SRZ2
SJX61913.1 Sporisorium reilianum SRS1
g943 Ustilago esculenta
XM_011389466.1 Ustilago maydis 521
MK756114.1 Sporisorium scitamineum
XM_029884498.1 Sporisorium graminicola
CP060301.1 Anthracocystis panici-leucophaei
0.1
B
CP094211.1 Ustilago hordei
g4344 Ustilago esculenta
CBQ73540.1 Sporisorium reilianum SRZ2
XM_014801567.1 Moesziomyces antarcticus
SAM82529.1 Ustilago bromivora
XM_011391385.1 Ustilago maydis 521
XM_029886529.1 Sporisorium graminicola
CDR88350.1 Sporisorium scitamineum
CP060308.1 Anthracocystis panici-leucophaei
SJX63374.1 Sporisorium reilianum SRS1
2
C
XP_016292642.1 Kalmanozyma brasiliensis
XP_012189864.1 Pseudozyma hubeiensis
QJS39920.1 Sporisorium scitamineum
g1072 Ustilago esculenta
XP_02973906.1 Sporisorium graminicola
SAM80652.1 Ustilago bromivora
CBQ71904.1 Sporisorium reilianum SRZ2
SJX61775.1 Sporisorium reilianum SRS1
CP060301.1 Anthracocystis panici-leucophaei
UTT94130.1 Ustilago hordei
1

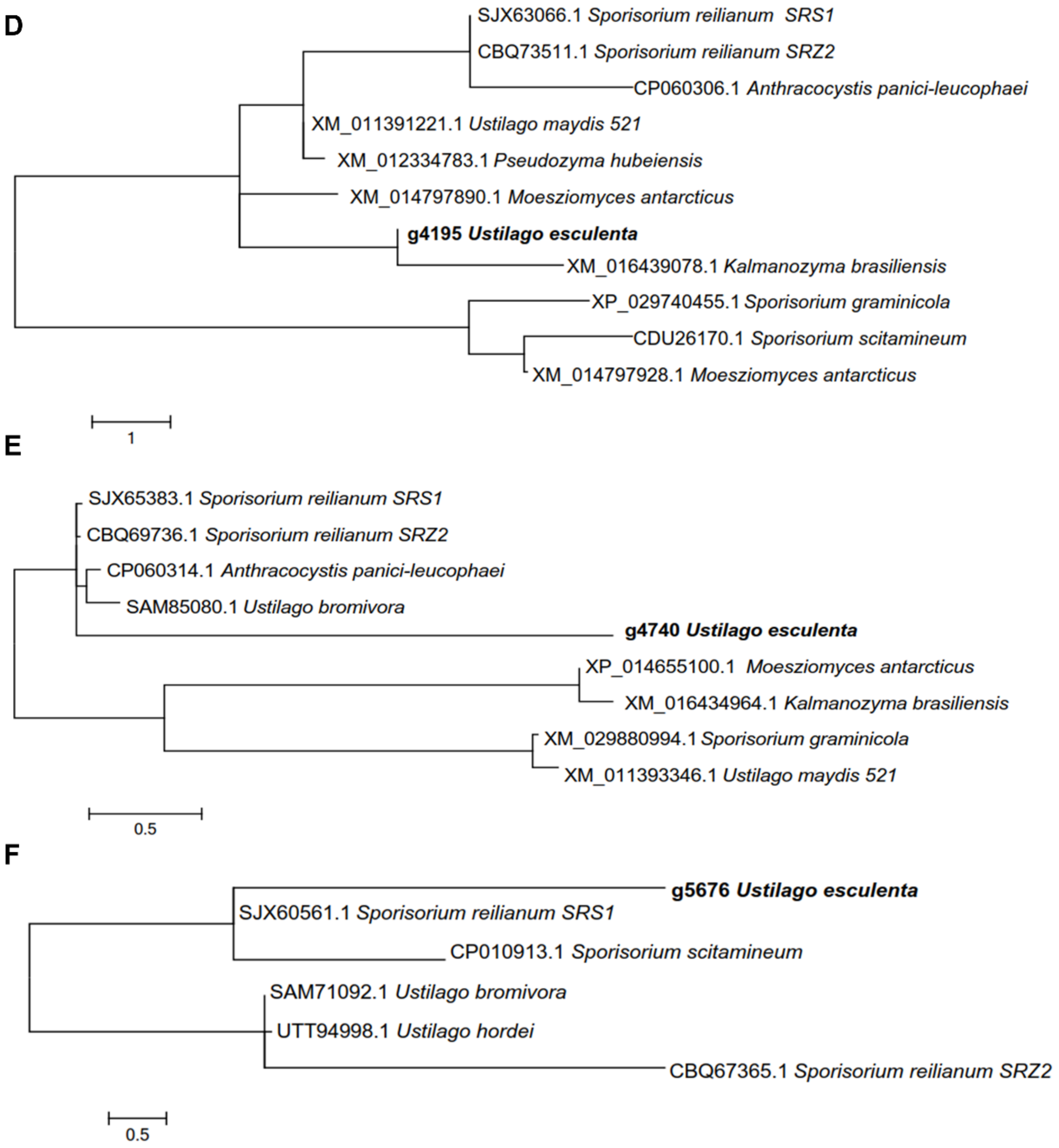

Figure S6. Phylogenetic analysis of putative secretory proteins. Homologs of secreted proteins were identified by searching against the NR database (NCBI). The phylogenetic trees were constructed in MEGA 7.0 by using the maximum-likelihood, in which bootstrap replications was 1000 times, and the model used was Jones-Taylor-Thornton (JTT) model.

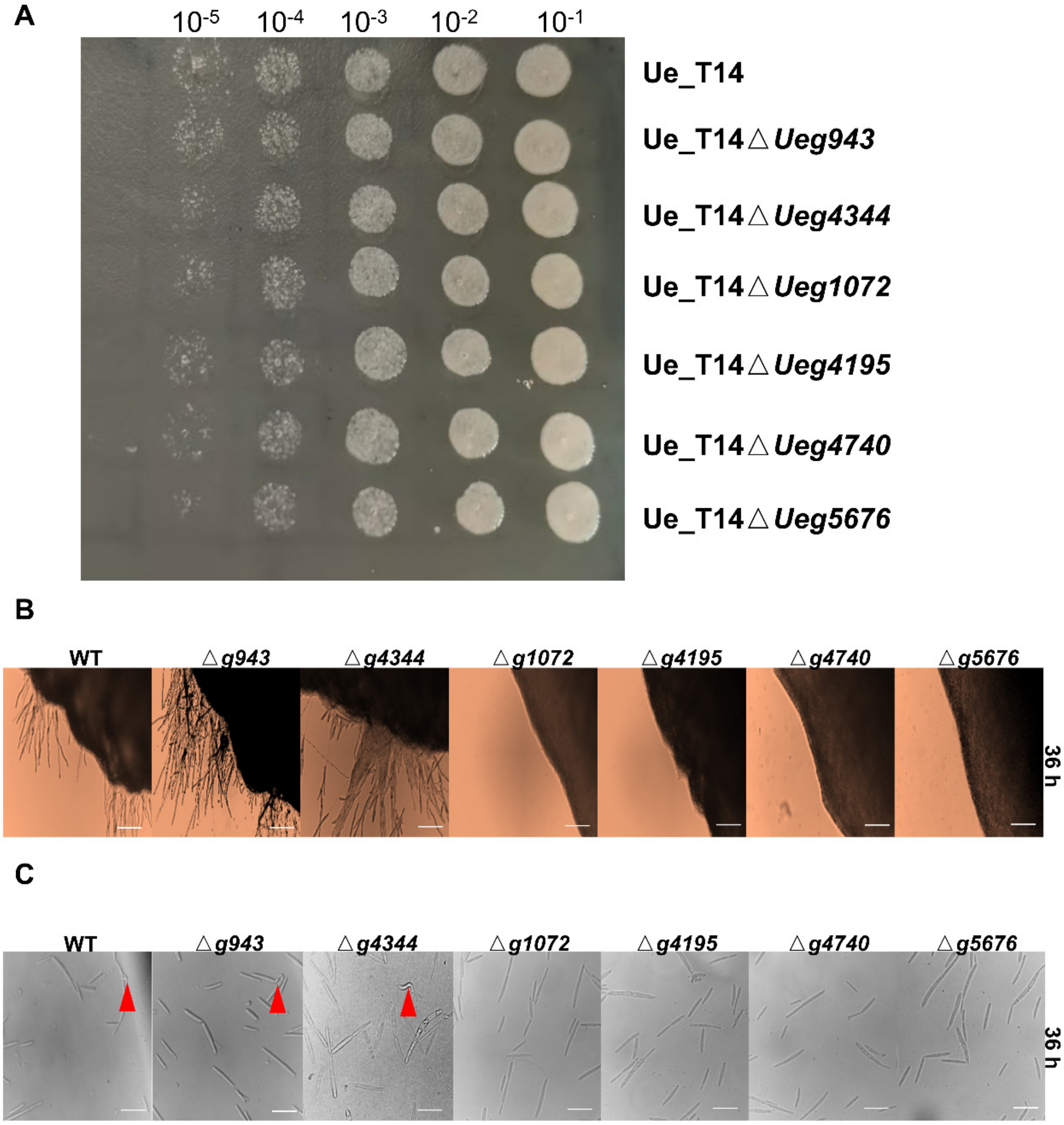

Figure S7. Effects of *g943*, *g4344*, *g1072*, *g4195*, *g4740*, *g5676* deletion on haploid growth, mating a nd filamentous growth. They were spotted onto YEPS medium. UeT14Δ*g943* and UeT55Δ*g943* cro ss,UeT14Δ*g4344* and UeT55Δ*g4344* cross,UeT14Δ*g1072* and UeT55Δ*g1072* cross,UeT14Δ*g4195* and U eT55Δ*g4195* cross,UeT14Δ*g4740* and UeT55Δ*g4740* cross,UeT14Δ*g5676* and UeT55Δ*g5676* cross (A)D eletion of *g943*, *g4344*, *g1072*, *g4195*, *g4740 and g5676* didn't impair haploid growth. Photograph the 7 2 h haploid growth state with camera (B) Compared with WT, mating and filamentous growth of the gene deletion of *g943*, *g4344*, *g1072*, *g4195*, *g4740*, *g5676*. Deletion of *g943* and *g4344* didn't imp air mating and filamentous growth. Deletion of *g1072*, *g4195*, *g4740* and *g5676* impair mating and filamentous growth. Photograph the 36 h filamentous growth state with light microscope. The scal e bar represents 100 μm in 36 h. (C) Compared with the WT type, forming state of the conjugatio n tube of the gene deletions of *g943*, *g4344*, *g1072*, *g4195*, *g4740*, and *g5676* at 36 h. Deletion of *g9 43* and *g4344* didn't impair forming of the conjugation tube. Deletion of *g1072*, *g4195*, *g4740* and *g 5676* impair forming of the conjugation tube. (forming of conjugation tube pointed by red arrow). Photograph the 36 h conjugation tube state with light microscope. The scale bar represents 62.5 μ

m in 36 h.

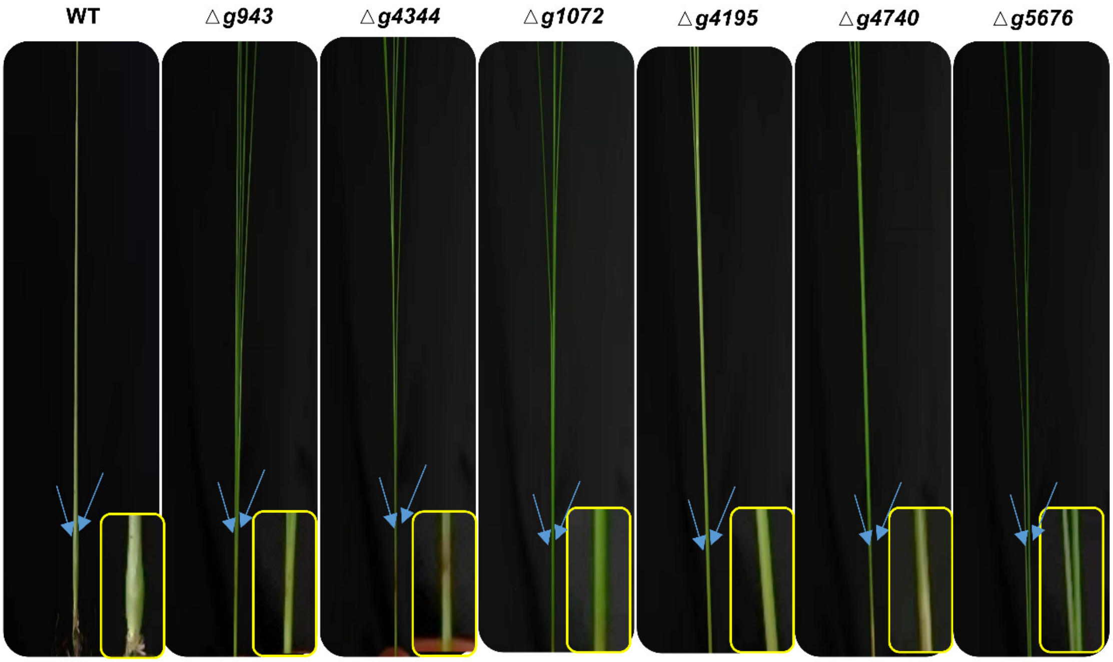

Figure S8. Phenotypes of the swollen stems. Samples infected with the wild-type strains UeT14 × UeT55 (left) and the corresponding Δ*g943* (UeT14Δ*g943* × UeT55Δ*g943*), Δ*g4344* (UeT14Δ*g4344* × UeT55Δ*g4344*), Δ*g1072*(UeT14Δ*g1072* × UeT55Δ*g1072*), *g4195*(UeT14Δ*g4195* × UeT55Δ*g4195*), Δ*g4740*(UeT14Δ*g4740* × UeT55Δ*g4740*), Δ*g5675* (UeT14Δ*g5676* × UeT55Δ*g5676*)mating strains (right) were collected at 90 dpi. The yellow box at the lower right corner magnifies the stem swelling phenotype.

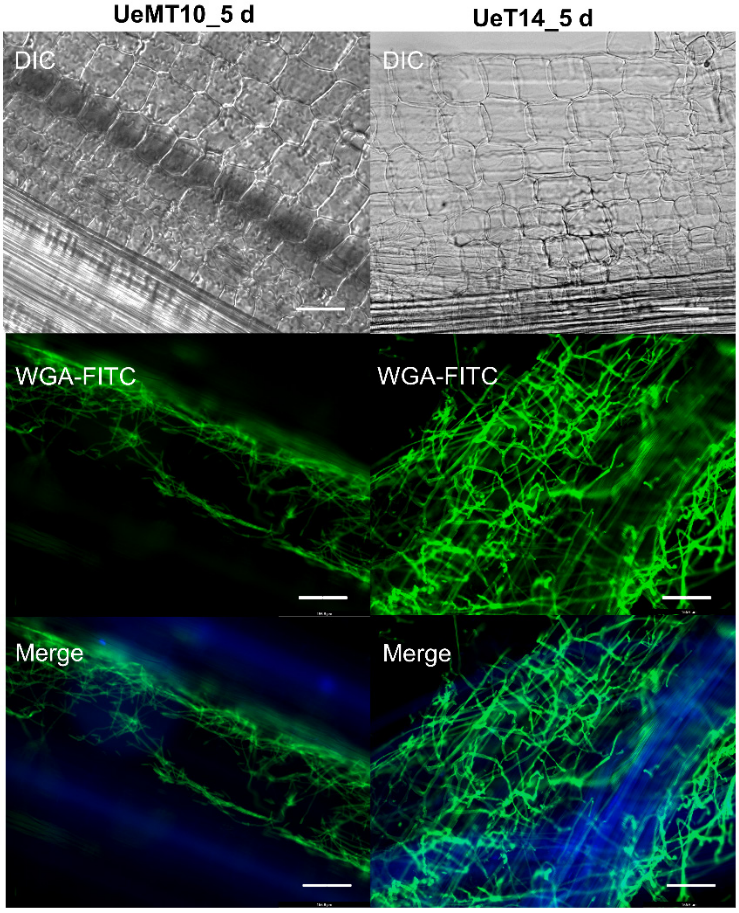

Figure S9. The phenotypes of haploids on plant surface. Photomicrographs were taken 5 dpi post infection (dpi). The UeT14 strain and UeMT10 strain do not induce callose deposition. The callose was stained by Aniline blue. It is excited at 405 nm, and the fluorescence was detected at 440 to 460 nm. The mycelium of *U. esculenta* is stained by WGA-FITC. It is excited at 488 nm, and the fl uorescence was detected at 495 to 530 nm. Plant cell structure is detected by DIC. Merge image s hows the superimposition of mycelium display image and aniline blue deposition image. Scale bar represents 62.5 μm.
